# Jun-*APOE*-LRP1 axis promotes tumor metastasis in colorectal cancer

**DOI:** 10.17305/bb.2023.9248

**Published:** 2023-12-01

**Authors:** Lingyuan He, Mengchen Shi, Shuwei Ren, Jingdan Zhang, Yu Tian, Xiangling Yang, Huanliang Liu

**Affiliations:** 1 Department of Clinical Laboratory, The Sixth Affiliated Hospital, Sun Yat-sen University, Guangzhou, Guangdong, China; 2 Department of General Surgery, The Sixth Affiliated Hospital, Sun Yat-sen University, Guangzhou, Guangdong, China; 3 Guangdong Provincial Key Laboratory of Colorectal and Pelvic Floor Diseases, Guangdong Institute of Gastroenterology, The Sixth Affiliated Hospital, Sun Yat-sen University, Guangzhou, Guangdong, China

**Keywords:** Apolipoproteins, colorectal cancer (CRC), transcription factor, lipoprotein receptor (LRP), metastasis

## Abstract

Apolipoprotein E (apoE) has previously been reported to play vital roles in tumor progression. However, the impact of apoE on colorectal cancer (CRC) metastasis remains largely unexplored. This study aimed to investigate the role of apoE in CRC metastasis and to identify the transcription factor and receptor of apoE involved in the regulation of CRC metastasis. Bioinformatic analyses were conducted to examine the expression pattern and prognosis of apolipoproteins. *APOE*-overexpressing cell lines were utilized to explore the effects of apoE on proliferation, migration, and invasion of CRC cells. Additionally, the transcription factor and receptor of apoE were screened via bioinformatics, and further validated through knockdown experiments. We discovered that the mRNA levels of *APOC1*, *APOC2*, *APOD*, and *APOE* were higher in the lymphatic invasion group, and a higher *APOE* mRNA level indicated poorer overall survival and progression-free interval. In vitro studies demonstrated that *APOE*-overexpression did not affect proliferation but promoted the migration and invasion of CRC cells. We also reported that *APOE*-expression was modulated by the transcription factor Jun by activating the proximal promoter region of *APOE*, and *APOE*-overexpression reversed the metastasis suppression of *JUN* knockdown. Furthermore, bioinformatics analysis suggested an interaction between apoE and low-density lipoprotein receptor-related protein 1 (LRP1). *LRP1* was highly expressed in both the lymphatic invasion group and the *APOE*^High^ group. Additionally, we found that *APOE*-overexpression upregulated LRP1 protein levels, and *LRP1* knockdown attenuated the metastasis-promoting function of *APOE*. Overall, our study suggests that the Jun-*APOE*-LRP1 axis contributes to tumor metastasis in CRC.

## Introduction

Despite tremendous advances in diagnosis and treatment, colorectal cancer (CRC) still ranks third in incidence and is the second leading cause of cancer-related death worldwide [1]. As a multistage and multifactorial process, CRC metastasis remains a major cause of high mortality and poor prognosis. Recent studies have reported that abnormal lipid metabolism is involved in the progression of CRC, and many genes related to lipid metabolism have been found to play an oncogenic role [2–6]. Apolipoprotein E (apoE) is a secreted protein involved in lipid metabolism and in the development of Alzheimer’s disease and atherosclerosis [7, 8]. Moreover, its additional role in tumor progression has been revealed. Zheng et al. [9] reported that apoE encapsulated in exosomes from tumor-associated macrophages promoted gastric cancer cell migration, indicating the protumoral role of apoE. In contrast, Pencheva et al. [10] identified three miRNAs targeting apoE as factors contributing to melanoma metastasis, suggesting that apoE is a metastasis suppressor. Evidently, apoE is likely to play different roles in different tumors. In recent years, abnormally high expression of apoE has been detected in the serum and tumor tissues of CRC patients [11, 12]. However, the regulation of CRC metastasis and progression by apoE and the underlying mechanisms still remain unclear.

*APOE* transcription is regulated by transcriptional regulatory elements, such as the proximal promoter, proximal enhancer, and distal enhancer, which contain binding sites for transcription factors [13]. It has been reported that cerebellar zinc finger protein 1 and 2 can bind to upstream regulatory element 1 (URE1), while upstream stimulator 1 and BK viral enhancer factor 1 can bind to URE3. In addition, liver-X-nuclear hormone receptor (LXR) and retinoid X receptor can bind to multi-enhancers 1 and 2. In addition, transcription factors, such as activating proteins 1 and 2, and nuclear factor-κB can also regulate the expression of apoE [13]. The transcriptional regulation of *APOE* has its specificity in relation to tissues and cells. Trusca et al. [14] showed that repressors can inhibit transcription of *APOE* in hepatocytes after binding of glucocorticoids to glucocorticoid elements in the *APOE* promoter, but not in macrophages. Notably, *APOE* transcription in neuronal cells cannot translate the splice variant *APOE-I3* formed by retention of intron 3, but cytokines secreted by astrocytes under stress can promote the conversion of *APOE-I3* to normal *APOE* mRNA [15]. In CRC cells, the transcriptional regulation of *APOE* is not yet known.

It has been reported that apoE binds to a group of proteins known as the low-density lipoprotein receptor (LDLR) family, which mainly includes the LDLR, the very-LDLR (VLDLR), and LDLR-related proteins (LRPs) [16]. As one of the subtypes of LRPs, lipoprotein receptor-related protein 1 (LRP1) is a mono-glycosylated protein composed of 4544 amino acids and has a molecular weight of approximately 600 kDa. In the presence of furin, LRP1 was cleaved into an extracellular subunit of 515 kDa and a transmembrane subunit of 85 kDa [17]. LRP1 is essential for early embryonic development, and knockout of *LRP1* was found to have lethal effects in mice [18]. In addition, brain-specific knockout of *LRP1* significantly impaired synaptic signaling in the nervous system [19]. LRP1 has also been shown to be involved in cell adhesion and motility. Wujak et al. [20] reported that LRP1 can interact with integrin-β1 and promote the activation and subsequent degradation of integrin-β1, thereby promoting cell migration. Another study showed that plasminogen activator inhibitor-1 produced by adipose stromal cells can interact with LRP1 and inhibit the connection between TGF-β/SMAD4 and cell adhesion complexes, promoting the development of obesity-related endometrial cancer [21]. Whether LRP1 is involved in the process of CRC metastasis remains unclear.

Here, we investigated the expression pattern and prognostic value of *APOE* in CRC using online databases. We also investigated the effects of *APOE* on proliferation and metastasis of CRC cells in vitro. In addition, we identified the transcription factor that activates the expression of *APOE* and the receptor that mediates the metastasis-promoting function of apoE.

## Materials and methods

### Online databases

The expression profiles and corresponding clinical characteristics of the indicated genes (*APOE*, *JUN*, and *LRP1*) in CRC samples were downloaded from UCSC Xena (https://xenabrowser.net/heatmap/). Data for the assay for targeting accessible chromatin with high-throughput sequencing (ATAC-seq) of colon adenocarcinoma were downloaded from The Cancer Genome Atlas (TCGA) data portal (https://gdc.cancer.gov/about-data/publications/ATACseq-AWG). Chromatin immunoprecipitation sequencing (ChIP-seq) data of H3K27ac, H3K4me3, and *JUN* in CRC cell lines were obtained from Cistrome DB (http://cistrome.org/db//#/). The interactions between apoE and the potential receptors were predicted from the STRING database (https://cn.string-db.org/cgi/input?sessionId=be2jwlEbiqJ1&input/_page/_show/_search=on).

### Construction of *APOE*-overexpressing stable cell lines and cell culture

Human colon cancer cell lines HCT116 and HCT8 (American Type Culture Collection, USA) were maintained in RPMI 1640 (Gibco, USA) supplemented with 10% fetal bovine serum (FBS, Gibco) and 1% penicillin/streptomycin (Gibco). All cells were cultured at 37 ^∘^C and 5% CO_2_. HCT116 and HCT8 cells were infected with *APOE-*overexpression lentivirus and vector control lentivirus (Shanghai Genechem Co., Ltd., China). The infected cells were probed with an appropriate concentration of puromycin (Gibco) for two weeks. Quantitative polymerase chain reaction (qPCR) and western blot were used to evaluate the efficiency of overexpression of apoE.

### Cell counting kit-8 (CCK-8) and colony formation assay

Cells were seeded in 96-well plates (5×10^3^ cells/well), and proliferation ability was assessed using a CCK-8 kit (KeyGEN, China) at the indicated time points (0 h, 24 h, 48 h, 72 h, and 96 h). For the colony formation assay, 1×10^3^ cells were seeded in 6-well plates. After 14 days, the culture medium was removed, and the cells were washed three times with phosphate-buffered saline and fixed in methanol for 10 min. Cells were then stained with crystal violet solution (Beyotime, China) for 10 min. Colonies were counted using ImageJ.

### Transwell assay

Tumor cell migration and invasion were studied by transwell assay using 24-well Boyden chambers (Falcon, USA) with polycarbonate membranes with a pore size of 8.0 µm with or without Matrigel. In brief, 2×10^5^ tumor cells were seeded into the upper chamber, while the culture medium supplemented with 10% FBS was added to the lower chamber. After incubation for the appropriate time, the migrated or invaded cells were fixed with methanol, stained with crystal violet, and photographed under the microscope. The migrated and invaded cells were counted using ImageJ.

### siRNAs and plasmids transfection

The siRNAs targeting *LRP1* and *JUN* and the negative controls were synthesized by RiboBio (China). Plasmids pLV-*APOE* and pLV-Vector were purchased from Shanghai Genechem Co., Ltd., while pcDNA3.1-*JUN*, pcDNA3.1-Vector, and pGL4.10-*APOE* were purchased from WZ Biosciences Inc. (China). Renilla luciferase reporter plasmid pRL-TK was properly stored by our laboratory. Tumor cells were transfected with siRNAs or plasmids using Lipofectamine^®^ RNAiMAX or LipofectamineTM 3000 (Invitrogen, USA) according to the manufacturer’s instructions. The sequences of siRNAs were as follows: si-*LRP1*-1 sense: GUUUCGAGGUGGUGAUUCATT; si-*LRP1*-1 antisense: UGAAUCACCACCUCGAAACTT; si-*LRP1*-2 sense: GAGGAACCGUUUCUGAUCUTT; si-*LRP1*-2 antisense: AGAUCAGAAACGGUUCCUCTT; si-*JUN*-1 sense: UCUACGCAAACCUCAGCAATT; si-*JUN*-1 antisense: UUGCUGAGGUUUGCGUAGATT; si-*JUN*-2 sense: UCAUGCUAACGCAGCAGUUTT; si-*JUN*-1 antisense: AACUGCUGCGUUAGCAUGATT.

### Luciferase reporter assay

The firefly luciferase plasmid pGL4.10-*APOE* covers the promoter region of *APOE* (from −2000 bp of the transcription start site to +100 bp). The Renilla luciferase reporter plasmid pRL-TK was used as a reference control. A total of 1 × 10^4^ tumor cells were seeded in a 96-well plate and transfected with 100 ng pGL4.10-*APOE*, 100 ng pcDNA3.1-*JUN* (or pcDNA3.1 vector), and 10 ng pRL-TK. At 24 h after transfection, luciferase activity was determined using a Dual-Luciferase Assay System (Promega, China) according to the manufacturer’s instructions.

### Quantitative polymerase chain reaction (qPCR)

Total RNA was extracted from cells using an RNA Quick Purification Kit (ES science, China) and transcribed into complementary DNA (cDNA) using the PrimeScript^TM^ RT reagent kit with cDNA Eraser (Takara, Japan). The cDNA was subjected to qPCR using TB Green Premix Ex Taq (Takara). *GAPDH* was used as a reference control. The sequences of primers were as follows: human *APOE*-forward 5’-GGTCGCTTTTGGGATTACCT-3’ and reverse 5’-CCTTCAACTCCTTCATGGTCTC-3’; human *LRP1*-forward 5’-CTATCGACGCCCCTAAGACTT-3’ and reverse 5’-CATCGCTGGGCCTTACTCT-3’; human *JUN*-forward 5’-AACAGGTGGCACAGCTTAAAC-3’ and reverse 5’-CAACTGCTGCGTTAGCATGAG-3’; human *GAPDH*-forward 5’-GTCTCCTCTGACTTCAACAGCG-3’ and reverse 5’-ACCACCCTGTTGCTGTAGCCAA-3’.

### Western blot

Total protein was extracted from cells using RIPA buffer (Beyotime) containing protease and phosphatase inhibitors (KeyGEN). Cell lysates were centrifuged at 12000 *g* and 4 ^∘^C for 10 min. The supernatants were collected and measured using a BCA Protein Assay Kit, Pierce^®^ (Thermo, USA). Equal amounts of proteins mixed with 1 × loading buffer (Beyotime) were subjected to sodium dodecyl sulfate-polyacrylamide gel electrophoresis and transferred to 0.45 µm polyvinylidene fluoride membranes (Millipore, USA). After blocking with 5% bovine serum albumin (Biotopped, China) at room temperature for 1 h, the membranes were incubated with primary antibodies overnight at 4 ^∘^C, followed by incubation with secondary antibodies for 1 h at room temperature. Finally, a chemiluminescence kit (Meilunbio, China) was used to detect immunoreactive protein bands. The primary and secondary antibodies are as follows: anti-apoE (1:1000, 13366S; Cell Signaling Technology, USA), anti-c-Jun (1:1000, 9165S; Cell Signaling Technology), anti-p-c-Jun (1:1000, ab32385; Abcam, UK), anti-LRP1 (1:1000, DF2935-50; Affinity, China), anti-β-actin (1:5000, 66009-1-Ig-100UL; Proteintech, China), anti-GAPDH (1:5000, 10494-1-AP; Proteintech), anti-mouse IgG (1:5000, E030110-01; Earthox, China), anti-rabbit IgG (1:5000, E030120-01; Earthox).

### Enzyme-linked immunosorbent assay (ELISA)

Intracellular and extracellular apoE levels of tumor cells were determined using a commercial ELISA kit (Meimian, China) according to the manufacturer’s instructions. Absorbance values of the samples were measured at 450 nm within 10 min after completion of the reaction.

### Ethical statement

This manuscript does not contain any studies with human participants or animals performed by any of the authors.

### Statistical analysis

Two-sample *t*-tests and Fisher’s exact tests were used to compare two independent groups. Survival functions were evaluated with the Kaplan–Meier plotter and compared with the log-rank test. Statistical analyses were performed with GraphPad Prism 8.0 software. A *P* value of less than 0.05 was considered significant.

## Results

### *APOE* is overexpressed in colorectal (CRC) patients with lymphatic invasion and indicates poor prognosis

**Figure 1.**
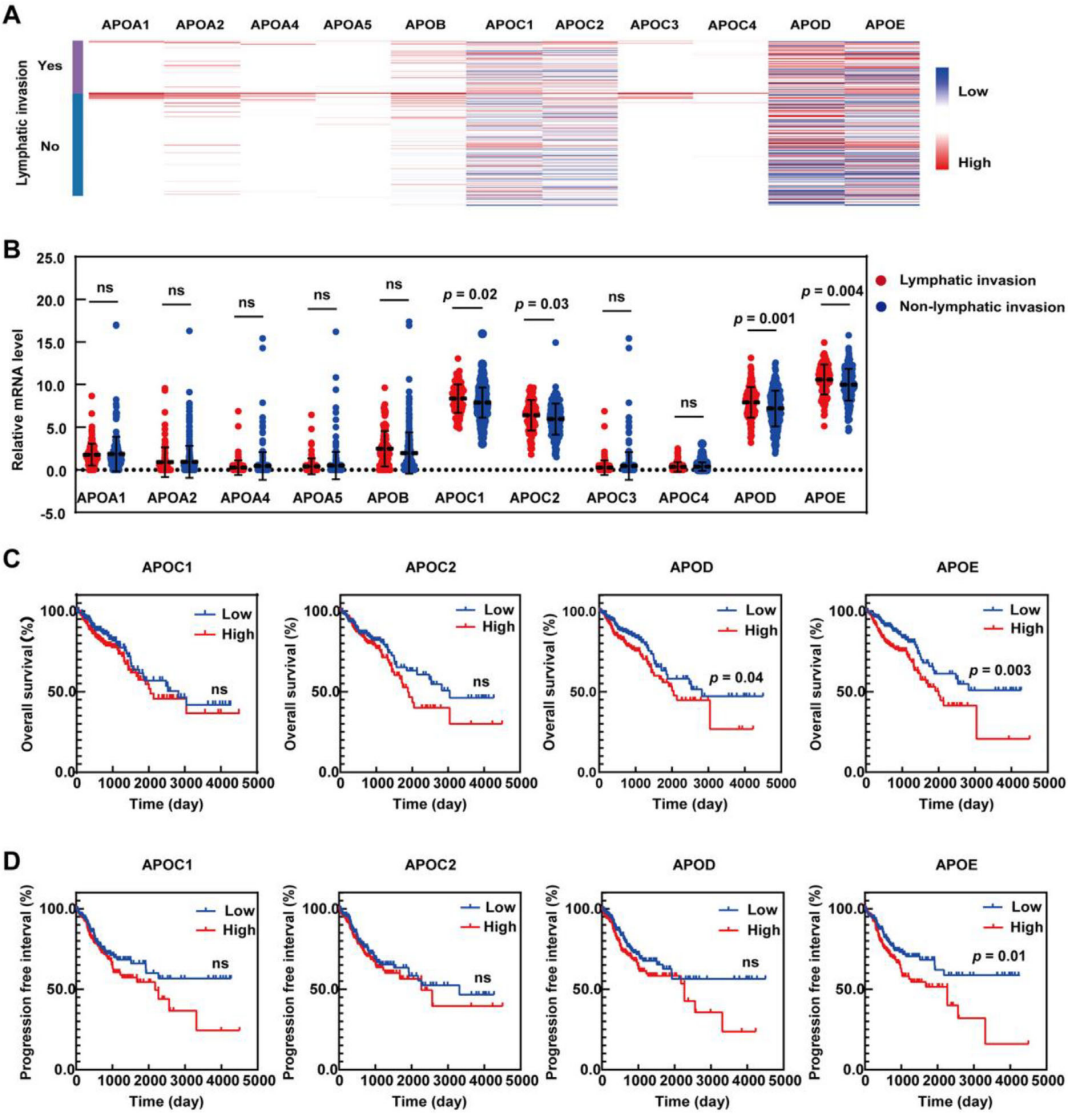
***APOE* is overexpressed in CRC patients with lymphatic invasion and indicates poor prognosis.** (A) Heatmap of apolipoproteins mRNA levels in CRC samples with or without lymphatic invasion; (B) Comparison of apolipoproteins mRNA levels between CRC samples with or without lymphatic invasion. (C and D) Kaplan–Meier plots of the overall survival and progression-free interval based on the mRNA levels of *APOC1*, *APOC2*, *APOD* and *APOE*. Data were obtained from the UCSC Xena database. *APOA1*: Apolipoprotein A1; *APOA2*: Apolipoprotein A2; *APOA4*: Apolipoprotein A4; *APOA5*: Apolipoprotein A5; *APOB*: Apolipoprotein B; *APOC1*: Apolipoprotein C1; *APOC2*: Apolipoprotein C2; *APOC3*: Apolipoprotein C3; *APOC4*: Apolipoprotein C4; *APOD*: Apolipoprotein D; *APOE*: Apolipoprotein E; CRC: Colorectal cancer; *ns*: No significance.

To illuminate the molecular characteristics of apolipoproteins in CRC, we initially analyzed the expression pattern of 11 apolipoproteins (apolipoprotein A1 [*APOA1*], apolipoprotein A2 [*APOA2*], apolipoprotein A4 *[APOA4*], apolipoprotein A5 [*APOA5*], apolipoprotein B [*APOB*], apolipoprotein C1 [*APOC1*], apolipoprotein C2 [*APOC2*], apolipoprotein C3 [*APOC3*], apolipoprotein C4 [*APOC4*], apolipoprotein D [*APOD*], and *APOE*) in CRC patients with or without lymphatic invasion using the UCSC Xena database (Figure 1A). We found that *APOC1*, *APOC2*, *APOD*, and *APOE* mRNA levels were significantly higher in the group with lymphatic invasion than in the group without lymphatic invasion, whereas the mRNA levels of the remaining apolipoproteins were not significantly different between the two groups (Figure 1B).

To further determine the potential prognostic value of the apolipoproteins that were abnormally expressed in the lymphatic invasion group, we assessed the overall survival (OS) and progression-free interval (PFI) of CRC patients according to the mRNA level of each apolipoprotein using the Kaplan–Meier plotter. The OS of the high *APOD* and high *APOE* groups was shorter than that of the low *APOD* and low *APOE* groups (Figure 1C). However, it was found that only high *APOE* was associated with worse PFI (Figure 1D). Overall, these results suggest that *APOE* is a facilitating factor for the progression of CRC. Therefore, *APOE* was prioritized for further biological and mechanistic studies.

### *APOE* promotes CRC cell migration and invasion in vitro

**Figure 2.**
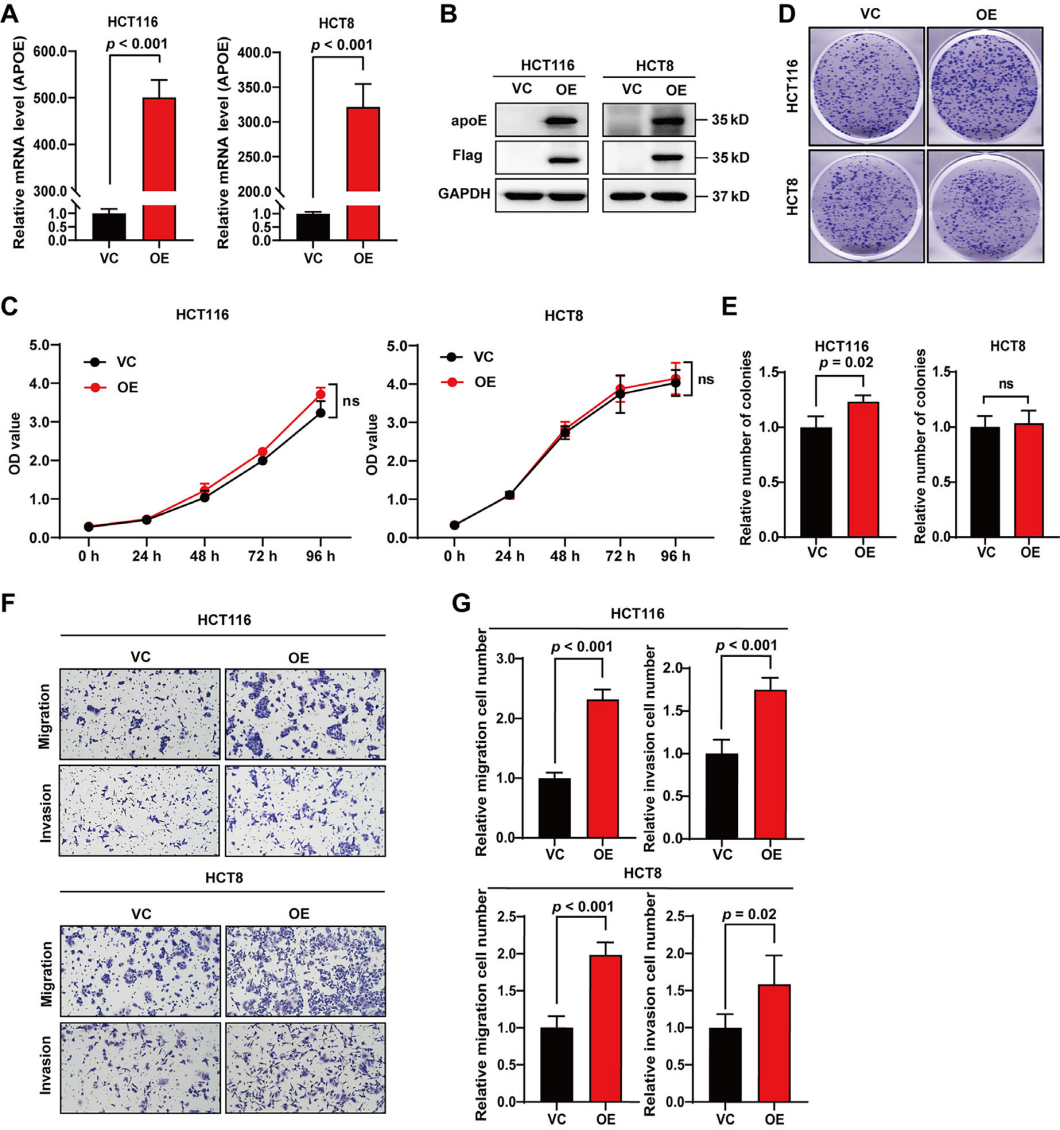
***APOE*-overexpression promotes CRC cell migration and invasion in vitro.** The efficiency of *APOE*-overexpression in HCT116 and HCT8 cells were validated by qPCR (A) and western blot (B); (C) The cell proliferation curves of HCT116 and HCT8 cells with or without *APOE*-overexpression evaluated by CCK-8 assay; (D) Representative images of colony formation assays; (E) Quantitative results of colony formation assays. The colony numbers were counted using ImageJ; (F) Representative images of migration and invasion assays of HCT116 and HCT8 cell lines in the presence or absence of *APOE-*overexpression; (G) Quantitative results of migration and invasion assays. The migrated and invaded cells were counted using ImageJ. apoE: Apolipoprotein E; CCK-8: Cell counting Kit-8; CRC: Colorectal cancer; OD: Optical density; OE: Overexpression; qPCR: Quantitative polymerase chain reaction; VC: Vector control; ns: No significance.

To investigate the effects of *APOE* on CRC, HCT116 and HCT8 cells were selected to generate stable *APOE*-overexpressing cell lines. The efficiency of *APOE*-overexpression was confirmed at both mRNA (Figure 2A) and protein (Figure 2B) levels. The CCK-8 assay showed that the cell proliferation rates of HCT116 and HCT8 cells were comparable between the control and overexpression groups (Figure 2C). Accordingly, the colony formation assay showed that the colony-forming ability of *APOE*-overexpressing HCT116 cells was significantly stronger than that of the control group, whereas there was no significant difference between the control and overexpression groups for HCT8 cells (Figure 2D and 2E). These results indicate that *APOE*-overexpression has almost no significant effect on the proliferation of HCT116 and HCT8 cells. We also performed a transwell assay to investigate the role of *APOE* on cell motility. Remarkably, the migrated and invaded cells of the overexpression groups were significantly higher than those of the control groups in both HCT116 and HCT8 cells (Figure 2F and 2G), indicating that overexpression of *APOE* promotes the migration and invasion of CRC cells.

### Jun activates *APOE*-expression in CRC cells

**Figure 3.**
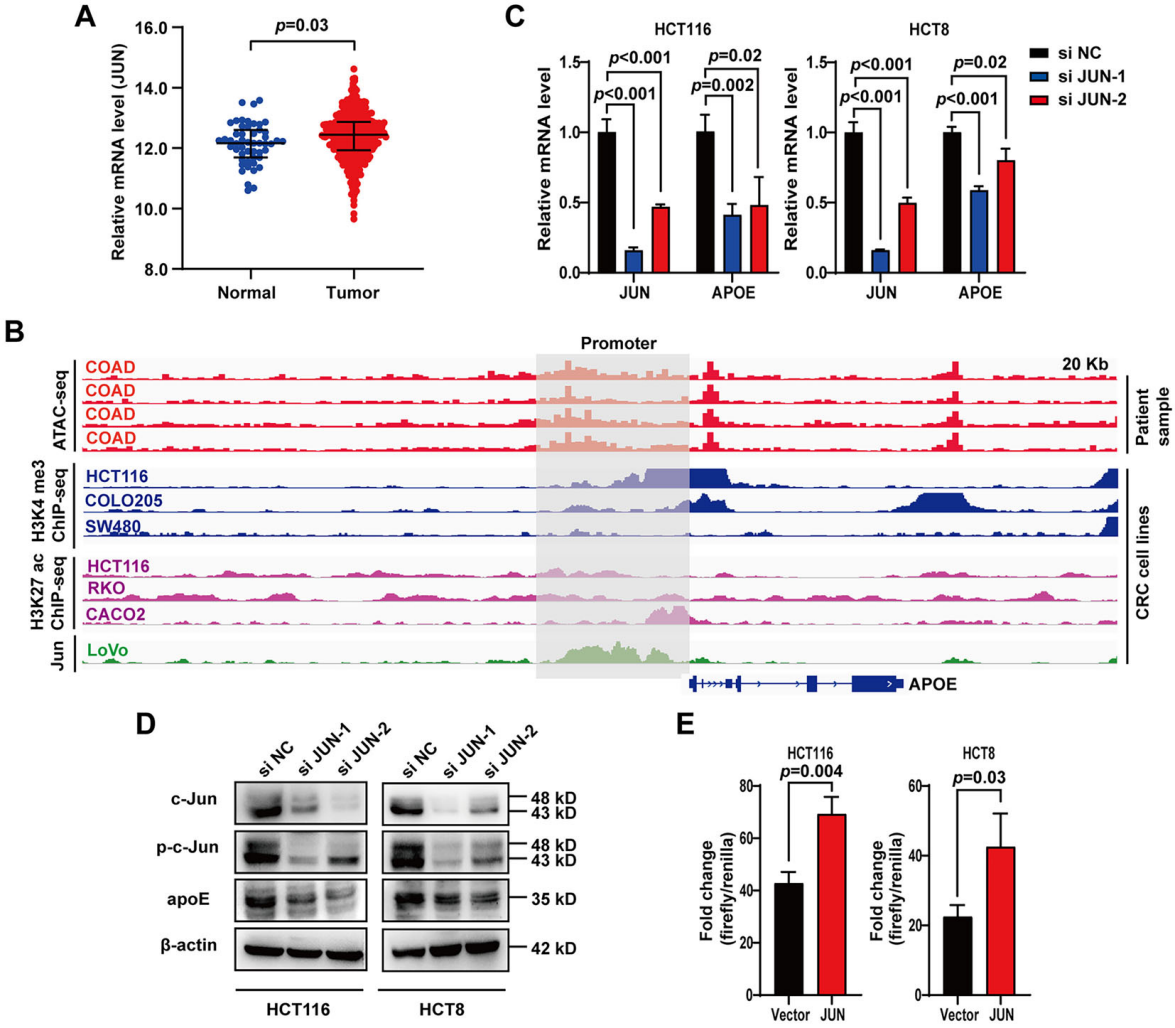
**Jun activates *APOE-*expression in CRC cells.** (A) Comparison of *JUN* mRNA levels between normal and tumor samples. Data were obtained from the UCSC Xena database; (B) Representative ATAC-seq and ChIP-seq signals in the *APOE* promoter region. The ATAC-seq data of CRC patients were downloaded from the TCGA data portal. ChIP-seq data for H3K4me3, H3K27ac, and *JUN* were downloaded from the Cistrome DB database; (C and D) qPCR and western blot were performed to determine the *APOE* mRNA and protein levels following knockdown of *JUN* in HCT116 and HCT8 cell lines; (E) HCT116 and HCT8 cells were cotransfected with pGL4.10-*APOE*, pcDNA3.1-*JUN* (or pcDNA3.1 vector), and pRL-TK, and the luciferase activity was determined 24 h post-transfection. Firefly luciferase/Renilla luciferase was used to indicate promoter activity. apoE: Apolipoprotein E; ATAC-seq: Assay for targeting accessible chromatin with high-throughput sequencing; ChIP-seq: Chromatin immunoprecipitation sequencing; COAD: Colon adenocarcinoma; CRC: Colorectal cancer; TCGA: The Cancer Genome Atlas; qPCR: Quantitative polymerase chain reaction.

Having clarified the biological significance of *APOE* in CRC, we sought to investigate the molecular basis of *APOE*-expression. Gene expression is strictly modulated by transcription factors. It has been previously reported that c-Jun encoded by *JUN* regulates *APOE* expression in hepatocytes and macrophages. Here, we investigated the transcriptional regulation of *APOE* expression by Jun in CRC cells. First, UCSC Xena data showed that tumor tissues had higher *JUN* mRNA levels than normal tissues (Figure 3A). Then, ATAC-seq data from TCGA showed that the promoter region of *APOE* in CRC samples had high chromatin accessibility (top 4 red tracks, Figure 3B). Further analysis of ChIP-seq data from the CRC cell lines showed that the promoter region of *APOE* had consistent H3K27 ac and H3K4 me3 signals (tracks 5–10, Figure 3B). As expected, we also found that Jun had readily detectable signals in the *APOE* promoter region (green track, Figure 3B).

To further clarify the transcriptional regulatory effect of Jun on *APOE*-expression, we knocked down *JUN* in HCT116 and HCT8 cells. We found that both mRNA and protein levels of *APOE* were significantly downregulated (Figure 3C and 3D). The level of p-c-Jun was also decreased when *JUN* was knocked down (Figure 3D). In addition, we examined the effect of *JUN* on the transcriptional activity of the *APOE* promoter using a dual-luciferase reporter assay. We found that the level of Firefly luciferase/Renilla luciferase was significantly higher in the *JUN*-overexpression group than in the control group (Figure 3E), indicating that *JUN* positively regulates *APOE*-expression.

### *APOE*-overexpression reverses the metastasis suppression of *JUN* knockdown

**Figure 4.**
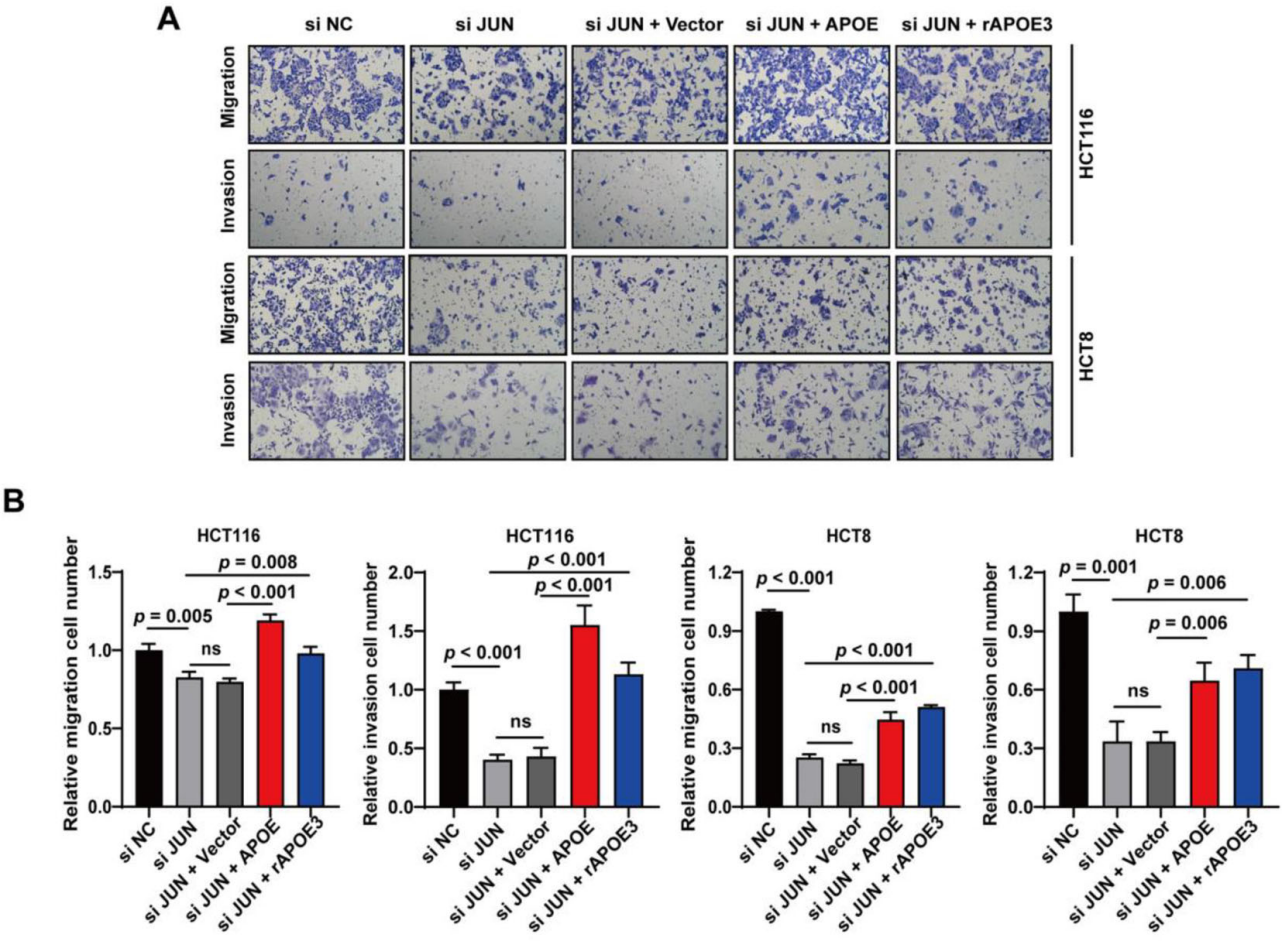
***APOE*-overexpression reverses the metastasis suppression of *JUN* knockdown.** (A) Following knockdown of *JUN*, HCT116, and HCT8 cells were transfected with pLV-*APOE* (pLV-Vector) or treated with 2 µg/mL r*APOE3*. Then, transwell assays were performed. Representative migration and invasion images are shown; (B) Quantitative results of transwell assays. The migrated and invaded cells were counted using ImageJ. *APOE*: Apolipoprotein E; r*APOE3*: Recombinant *APOE3*; ns: No significance.

The above findings suggest that *APOE* promotes CRC metastasis, but the regulation of Jun, which acts as a transcription factor of *APOE* on the migration and invasion of CRC cells, remains unknown. To clarify this, we performed transwell assays after knocking down *JUN* in HCT116 and HCT8 cells with siRNAs. We found that the migration and invasion abilities of HCT116 and HCT8 cells were significantly reduced after knockdown of *JUN* (Figure 4A and 4B). However, after transient transfection with *APOE*-overexpressing plasmids in HCT116 and HCT8 cells, the inhibitory effects on migration and invasion caused by *JUN* knockdown were partially or completely reversed. Moreover, treatment of HCT116 and HCT8 cells with 2 µg/mL recombinant *APOE3* (r*APOE3*) for 24 h also significantly reversed the inhibitory effects of *JUN* knockdown on migration and invasion. Overall, these results demonstrated that *APOE*-overexpression reversed the metastasis suppression caused by knockdown of *JUN*

### *LRP1* is highly expressed in CRC patients with lymphatic invasion and high *APOE* level, and *APOE*-overexpression upregulates LRP1 protein level

**Figure 5.**
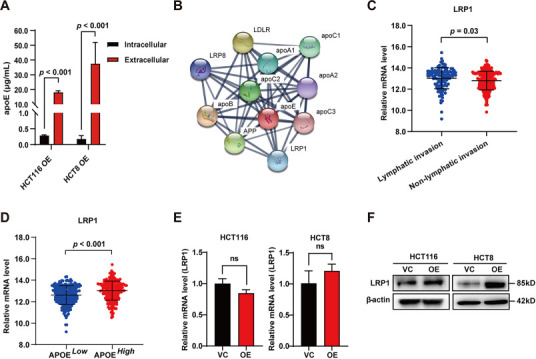
***LRP1* is highly expressed in CRC patients with lymphatic invasion and high *APOE* level, and *APOE*-overexpression upregulates LRP1 protein level.** (A) The intracellular and extracellular apoE levels of HCT116 and HCT8 cells with *APOE-*overexpression were determined by ELISA; (B) Schematic diagram of protein interactions of apoE. Data were obtained from the STRING database; (C) Comparison of *LRP1* mRNA levels between the lymphatic invasion group and the non-lymphatic invasion group; (D) Comparison of *LRP1* mRNA levels between the *APOE*^High^ group (median *APOE* mRNA level > 10.24) and *APOE*^Low^ group (median *APOE* mRNA level < 10.24). Data were obtained from the UCSC Xena database; (E) Relative *LRP1* mRNA levels of CRC cells with or without *APOE*-overexpression; (F) Determination of LRP1 protein levels in HCT116 and HCT8 cells with or without *APOE*-overexpression using western blot. apoA1: Apolipoprotein A1; apoA2: Apolipoprotein A2; apoB: Apolipoprotein B; apoC1: Apolipoprotein C1; apoC2: Apolipoprotein C2; apoC3: Apolipoprotein C3; apoE: Apolipoprotein E; APP: Amyloid precursor protein; CRC: Colorectal cancer; LDLR: Low-density lipoprotein receptor; LRP1: Lipoprotein receptor-related protein 1; LRP8: Lipoprotein receptor-related protein 8; OE: Overexpression; VC: Vector control; ns: No significance; ELISA: Enzyme-linked immunosorbent assay.

Because apoE is a secreted protein, we determined intracellular and extracellular levels of apoE in *APOE*-overexpressing CRC cells by ELISA. We found that the extracellular apoE level was much higher than the intracellular apoE level, indicating that most of the apoE was secreted from the cell (Figure 5A). Therefore, we suspected that there might be a putative receptor that mediates the metastasis-promoting function of apoE. To identify this putative receptor, we first used the online database STRING to predict the proteins that interact with apoE. Ten proteins with high combined scores were obtained, including six apolipoproteins and four transmembrane receptors (Figure 5B, Table 1). The interaction between amyloid precursor protein (APP) and apoE has been mainly reported to play a role in Alzheimer’s disease [22], while LDLR has been mainly associated with lipid transport [23] and LRP8 is an important receptor for signal transduction in the Reelin pathway [24]. It is worth noting that LRP1 is a multifunctional receptor that has been shown to be involved in the pathological processes of cancer [25]. Therefore, we focused on LRP1. Analysis of the UCSC Xena database showed that the *LRP1* mRNA level was significantly higher in the group with lymphatic invasion than in the group without lymphatic invasion (Figure 5C). In addition, the *LRP1* mRNA level of the *APOE*^High^ group (median *APOE* mRNA level > 10.24) was significantly higher than that of the *APOE*^Low^ group (median *APOE* mRNA level < 10.24) (Figure 5D). In addition, we determined the *LRP1* mRNA and protein levels of CRC cells with or without *APOE*-overexpression. The results showed that *APOE*-overexpression did not affect the mRNA level of *LRP1* (Figure 5E), but significantly increased the protein level (Figure 5F).

**Table 1:** Combined score of proteins interacting with apoE

**Apolipoprotein**		**Transmembrane receptor**	
**Protein**	**Combined score**	**Protein**	**Combined score**
apoB	0.996	LDLR	0.996
apoC2	0.993	APP	0.995
apoC1	0.992	LRP1	0.989
apoA1	0.991	LRP8	0.987
apoA2	0.985		
apoC3	0.984		

Combined score was analyzed using the STRING database. apoA1: Apolipoprotein A1; apoA2: Apolipoprotein A2; apoB: Apolipoprotein B; apoC1: Apolipoprotein C1; apoC2: Apolipoprotein C2; apoC3: Apolipoprotein C3; apoE: Apolipoprotein E; LDLR: Low-density lipoprotein receptor; APP: Amyloid precursor protein; LRP1: Lipoprotein receptor-related protein 1; LRP8: Lipoprotein receptor-related protein 8.

### *LRP1* knockdown suppresses the metastasis-promoting function of *APOE*

**Figure 6.**
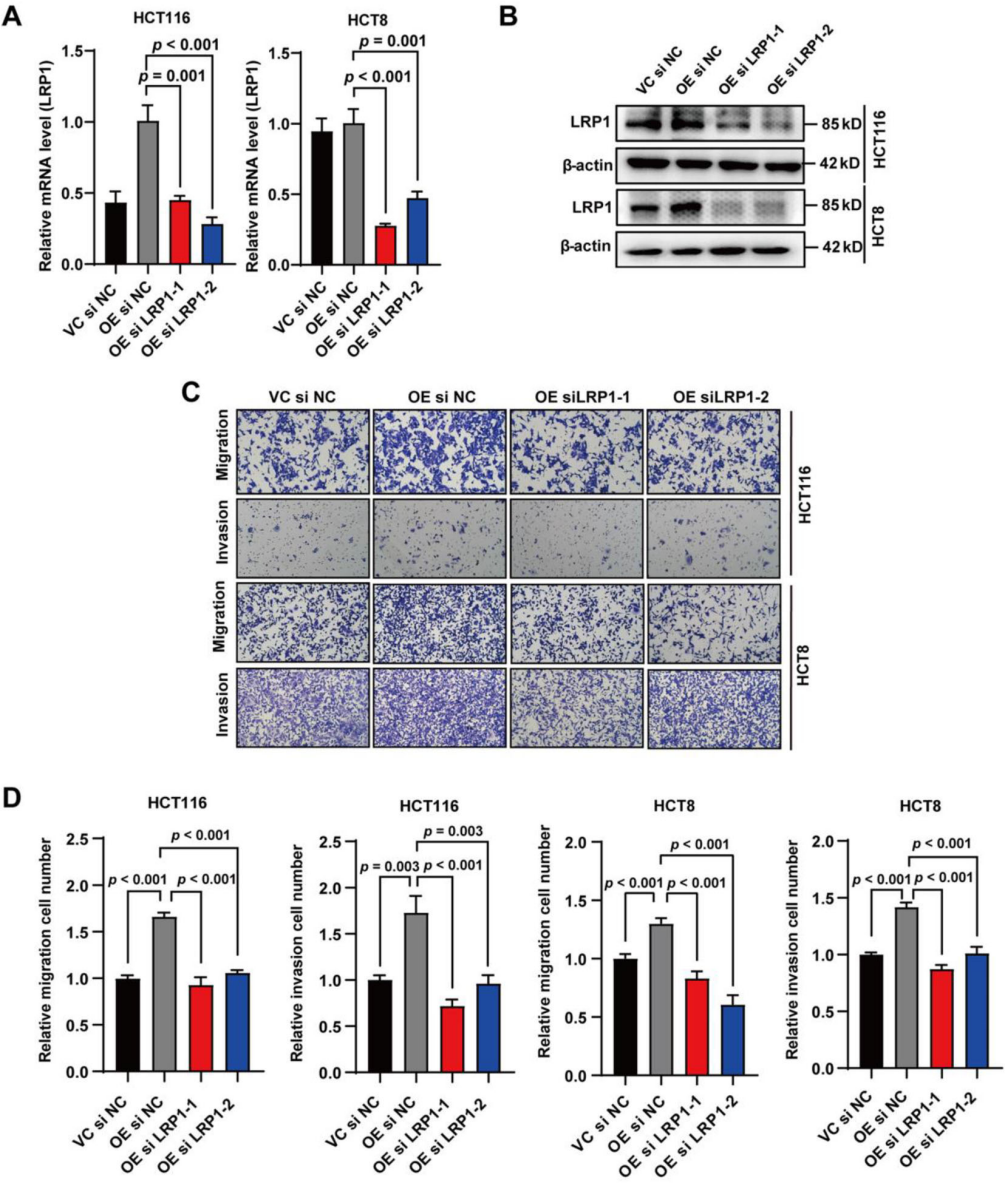
**LRP1 mediates the metastasis-promoting function of *APOE*.** HCT116 and HCT8 cells with *APOE*-overexpression were transfected with si-NC or si-*LRP1*, and the efficiency of *LRP1* knockdown was validated by qPCR (A) and western blot (B); (C) Transwell assays were performed 24 h post-transfection, and representative images of migration and invasion were shown; (D) Quantitative results of transwell assays. The migrated and invaded cells were counted using ImageJ. *APOE*: Apolipoprotein E; LRP1: Lipoprotein receptor-related protein 1; OE: Overexpression; qPCR: Quantitative polymerase chain reaction; VC: Vector control; ns: No significance.

To further investigate the effect of LRP1 on the metastasis-promoting function of *APOE*, we performed transwell assays after the knockdown of *LRP1* with siRNAs. The knockdown efficiency of *LRP1* was validated at both mRNA and protein levels. The *LRP1* mRNA and protein levels of the experimental groups were significantly lower than those of the control groups (Figure 6A and 6B). Subsequently, we found that the migration and invasion ability of HCT116 and HCT8 cells with *APOE*-overexpression was increased compared with control cells transfected with si-NC, which was consistent with the results in Figure 2F and 2G. However, when *APOE*-overexpressing CRC cells were transfected with two siRNAs targeting *LRP1*, the migrated and invaded cells of si-*LRP1* groups were significantly lower than those of si-NC groups, indicating that the migration- and invasion-promoting phenomenon of *APOE*-overexpression was significantly suppressed by *LRP1* knockdown (Figure 6C and 6D). All in all, these data clearly showed that LRP1 mediated the migration- and invasion-promoting function of *APOE*.

## Discussion

Reprogramming of lipid metabolism is one of the common metabolic features of tumor cells, which is closely associated with tumor metastasis and resistance to chemotherapy [26, 27]. Lipids are normally bound to apolipoproteins in the body to form lipoproteins. Apolipoproteins generally include apoA, apoB, apoC, apoD, and apoE, each of which can be divided into different subtypes. Here, we found that *APOC1*, *APOC2*, *APOD* and *APOE* mRNA levels were higher in the lymphatic invasion group, and the high levels of *APOD* and *APOE* indicated a poor prognosis.

Previous studies have also reported the association between apolipoproteins and CRC. A retrospective study of 508 CRC patients showed that patients with low levels of apoA1 had a worse prognosis than those with high levels of apoA1 [28]. Sirniö et al. [29] analyzed the association between serum apoA1 level and pathological tumor parameters, inflammatory markers, and survival prognosis in 144 CRC patients and found that apoA1 level was negatively correlated with tumor stage and inflammatory factors. The OS of patients with high apoA1 level is better than that of patients with low apoA1 level. Chen et al. [30] retrospectively analyzed the clinical data of 599 patients with advanced CRC and found that apoB/apoA1 ≥ 0.63 was an independent risk factor for poor survival in patients without liver metastases. Kopylov et al. [31] analyzed the protein expression profiles of serum samples from 41 healthy volunteers and 28 CRC patients and found that apoB levels differed significantly between the two groups. These studies suggest that apolipoproteins are abnormally expressed in CRC and their expression level is associated with the prognosis of CRC.

As an apolipoprotein, apoE is best known for its role in lipid metabolism. However, there is increasing evidence that it plays an important role in inflammation [32, 33], oxidative stress [34, 35], cell proliferation and angiogenesis [36], and immune response [37]. In addition, the role of apoE in tumor progression has been revealed, although it has been controversial. ApoE has been identified as an extracellular binding protein of leukocyte immunoglobulin-like receptor B4 (LILRB4), and disruption of the LILRB4/apoE interaction blocks the development of acute myeloid leukemia [38, 39]. Zheng et al. [9] found that apoE in tumor-associated macrophage-derived exosomes promoted gastric cancer metastasis. The effects of apoE on CRC progression remain unclear. In the present study, we found that *APOE* was overexpressed in advanced CRC patients and that high *APOE* mRNA level indicated poor prognosis, which was consistent with the findings of Zhao et al. [12]. In vitro experiments also demonstrated the metastasis-promoting role of *APOE*. However, *APOE* exerts tumor suppression roles in some cases. Lai et al. [40] reported that *APOE*-/- mice displayed collagen deposition in the peritoneal cavity, and the altered extracellular matrix in the peritoneal microenvironment accelerated the progression of ovarian cancer. In melanoma, LXR agonists robustly suppressed melanoma progression through the transcriptional induction of *APOE* [41]. We speculated that the opposite effects of *APOE* in tumor progression might be attributed to the complexity of metabolic characteristics and the tumor microenvironment in different cancer types.

Previous studies have shown that transcriptional regulation plays an important role in tumor progression [42, 43]. However, the regulation of *APOE* transcription, which is tissue-, differentiation-, and cell-specific, is still not clear in CRC. Here, we reported that the transcription factor Jun can interact with the proximal promoter region of *APOE* and upregulate its expression. Trusca et al. [44] showed that Jun could upregulate *APOE*-expression in hepatocytes but downregulate *APOE*-expression in macrophages. Combined with our results, these opposing effects of Jun on *APOE*-expression reflect the cell specificity of *APOE* transcription. Jun is a core member of the transcription factor complex AP-1, which is involved in the oncogenesis of various cancers [45]. In our study, we found that knocking down *JUN* suppressed the metastasis of CRC cells which was effectively reversed by *APOE*-overexpression and treatment with r*APOE3*. The tumor-promoting effect of *JUN* has also been described in other studies. Liu et al. [46] showed that the transcription factor Jun can inhibit the transcription of miR-22, an important tumor-suppressive miRNA. In a study by Xiang et al. [47], activation of Jun with hepatic leukemia factor promoted hepatocellular carcinoma development and sorafenib resistance. In addition to Jun, other transcription factors, such as Zic1/Zic2 and KLF4, are also responsible for APOE-expression [48, 49]. It is worth noting that *APOE* transcription involves an intricate network of many interacting regulatory elements that regulate *APOE*-expression in cell-specific ways to meet different biological requirements.

**Figure 7.**
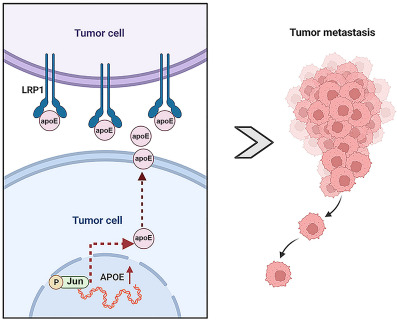
**Working model of the study.** Transcription factor Jun can activate the promoter of *APOE* and enhance its expression. The overexpressed apoE is secreted outside the cell and promotes the migration and invasion of CRC cells by binding to the LRP1 receptor. *APOE*: Apolipoprotein E; CRC: Colorectal cancer; LRP1: Lipoprotein receptor-related protein 1.

ApoE is a secreted protein that mainly binds to various receptors to exert different biological functions [8]. In this study, we found that most of apoE was secreted outside cells after its production, suggesting that apoE can exert its biological function in a paracrine manner. Through bioinformatic analysis and knockdown experiments, we identified LRP1 as the receptor that mediates the metastasis-promoting function of apoE. LRP1 is a multifunctional receptor that has been shown to be involved in many physiological and pathological processes, including cholesterol homeostasis, cell adhesion, and motility. Salama et al. [50] reported that tissue plasminogen activator promoted proliferation and migration of B16F10 melanoma cells, and this promoting function was mediated by LRP1. Sakamoto et al. [51] found that plasminogen activator inhibitor-1, derived from cancer-associated fibroblast-like cells, promoted the migration and invasion of esophageal squamous cell carcinoma cells and macrophages via LRP1. Another study identified the metastasis- and chemoresistance-promoting functions of the heat shock protein 90α-LRP1 signaling [52]. Our present findings have expanded the knowledge of LRP1/ligand interactions in regulating tumor progression.

Inevitably, there were some limitations in the present study. Our investigation of the role of *APOE* at CRC was based only on online database analysis and in vitro experiments, and the results of in vivo studies were not presented here. In addition, our study did not investigate whether other coreceptors are involved in mediating the metastasis-promoting function of apoE. Moreover, the underlying molecular mechanisms downstream of LRP1 remain to be elucidated. These shortcomings are precisely the direction of our future work.

## Conclusion

In conclusion, we identified apoE as a CRC metastasis-promoting factor. The abnormally high expression of *APOE* was transcriptionally regulated by Jun. Furthermore, we demonstrated that CRC cell-derived apoE exerts a migration- and invasion-promoting function via the LRP1 receptor in a paracrine manner. Our results revealed the metastasis-promoting function of the Jun-*APOE*-LRP1 axis in CRC (Figure 7). Thus, blocking the Jun-*APOE*-LRP1 axis could inhibit the metastasis of CRC cells. Overall, the findings obtained in the present study will help to better understand the pathophysiological mechanism of CRC progression and provide potential diagnostic biomarkers and targets for metastasis inhibition.
